# A Silent Fatal Presentation of Pulmonary Embolism: Reflection and Discussion

**DOI:** 10.7759/cureus.8813

**Published:** 2020-06-24

**Authors:** Qian Zhang, Zain U Abideen, Khine S Shan, Thomas Yoon, Muhammad Farooq

**Affiliations:** 1 Internal Medicine, Abington Hospital - Jefferson Health, Abington, USA; 2 Internal Medicine, University of Maryland Medical Center, Baltimore, USA

**Keywords:** pe, acute pulmonary embolism, pulmonary embolism (pe)

## Abstract

Acute pulmonary embolism is a common medical condition that clinicians face in practice. It is important to have a prompt diagnosis with proper management as it is associated with high morbidity and mortality. However, a timely diagnosis is often difficult to obtain especially when the presenting symptoms are atypical, but the consequence could be fatal. We present an 80-year-old gentleman who presented with a near-syncope episode who subsequently was found to have acute extensive bilateral pulmonary embolisms after a code blue event.

## Introduction

Pulmonary embolism (PE) is an important medical condition that could be related to high morbidity and mortality [1]. According to the Centers for Disease Control and Prevention (CDC), despite that the exact number of patients affected by PE is unknown, there are approximately 900,000 people in the United States diagnosed with PE annually. Moreover, there is around 60,000-100,000 annual death due to PE, with 10%-30% of the death occurs within one month after being diagnosed [2].

## Case presentation

Our patient is an 80-year-old gentleman presented to the emergency department (ED) after a sudden onset of a near-syncope episode accompanied by diaphoresis and lightheadedness while at rest. His pertinent medical history was significant for coronary artery disease (CAD) status post coronary artery bypass grafting (CABG), peripheral artery disease (PAD), hypertension, dyslipidemia, and chronic kidney disease (CKD). He was admitted to our hospital three months ago as his PAD was worsening that required left above the knee amputation and was subsequently discharged to the acute inpatient rehabilitation service. He subsequently recovered well and was discharged home after six weeks of rehabilitation. Emergency medical service (EMS) arrived at his house after the near-syncope episode without loss of consciousness, fall, chest pain, palpitation, dyspnea, fever, chills, nausea, vomiting, or bowel habit changes. Initial blood pressure was 80/40 mmHg and heart rate of 50 beats per minute. His symptoms had completely resolved upon EMS arrival, but it was determined to bring the patient to the ED for further evaluation.

**Figure 1:**
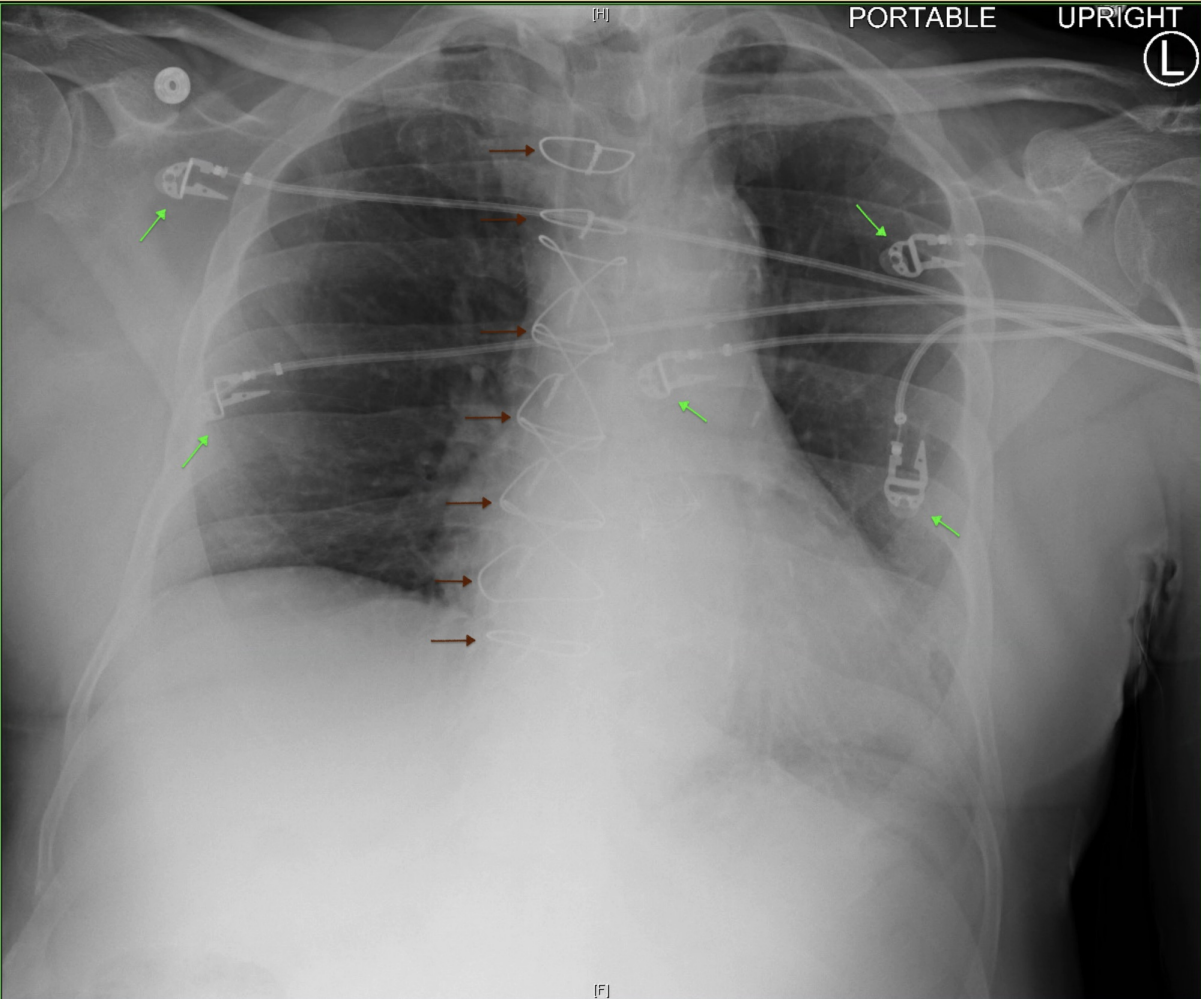
Chest X-Ray Normal size heart, no evidence of pulmonary vascular congestion, and negative for active pulmonary diseases. Presence of sternal cable wires from prior coronary artery bypass grafting (CABG) (red arrows) and electrocardiography (EKG) leads (green arrows). H: head, F: foot, L: left.

His vital signs in the ED were as follows: temperature 99.3°F, blood pressure 109/61 mmHg, heart rate 103 beats per minute, respiratory rate 18 breaths per minute and pulse oximetry saturation at 97% on room air. Physical examination was overall benign, and further review of systems was unremarkable. Initial laboratory findings were significant for lactic acid 3.0 mEq/L, creatinine 1.59 mg/dL, calculated glomerular filtration rate (GFR) 42 mL/minute, cardiac troponin <0.10 ng/mL, and cardiac b-type natriuretic peptide (BNP) 46 pg/mL. Electrocardiography (EKG) showed sinus tachycardia. Chest x-ray (CXR) showed normal size heart, no evidence of pulmonary vascular congestion, and negative for active pulmonary diseases (Figure 1). The patient’s episode of near syncope was thought to be related to hypovolemia or potential rate control medication side effects. He was given one liter of normal saline and was admitted to the general medicine floor for further observation. His creatinine and lactic acid levels normalized with fluid resuscitation. The patient remained to be hemodynamically stable overnight.

**Figure 2:**
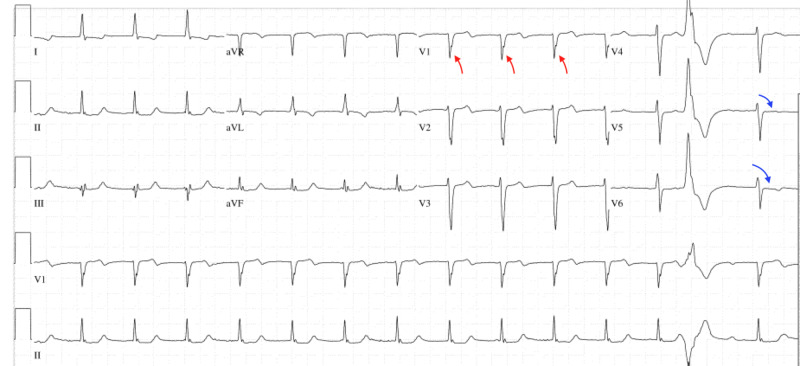
Electrocardiography Red arrows indicate a new-onset of incomplete left bundle branch block (LBBB). Blue arrows show ST&T wave abnormalities of the lateral leads.

On day 2 of hospitalization, a code blue was activated due to the sudden onset of pulseless electrical activity (PEA) arrest as the patient was found to be unresponsive in bed. He was hemodynamically stable and saturating well on room air prior to the code activation. He also denied chest pain, diaphoresis, or dyspnea. Emergent high-quality cardiopulmonary resuscitation (CPR) began according to the advanced cardiovascular life support (ACLS) protocol as the return of spontaneous circulation (ROSC) was later achieved. The patient was intubated, sedated, and transferred to the cardiac surgical unit (CSU) for further management. EKG during the code blue revealed findings that were concerning for anterolateral myocardial infarction (Figure 2). The patient was taken to the cardiac catheterization laboratory for an urgent exploration of the coronary arteries as troponin levels were trending upwards to possibly explain for the PEA arrest. Catheterization showed patent bypass grafts along with a hyperdynamic left ventricular systolic function. He remained to be stable overnight. 

**Figure 3:**
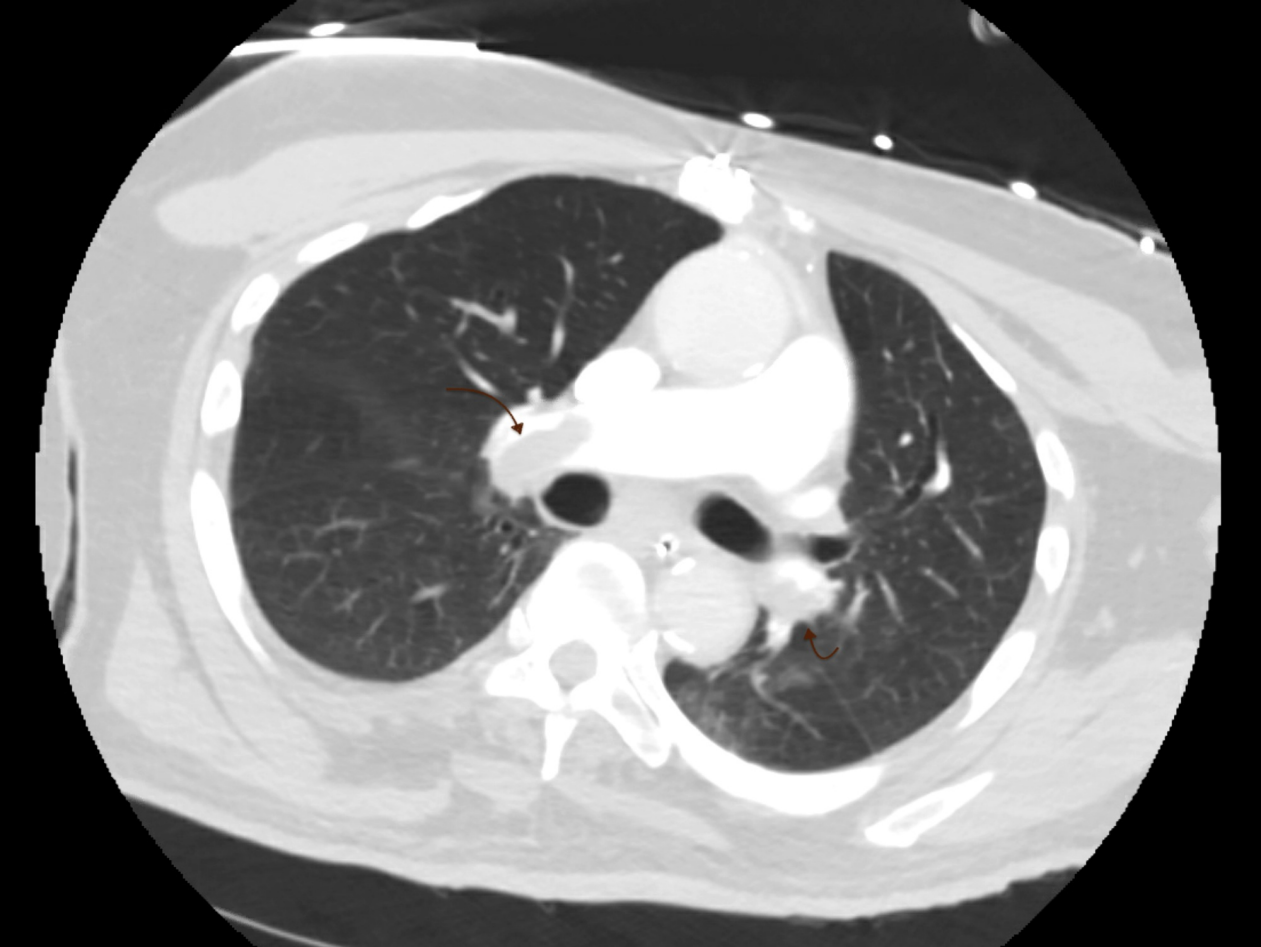
CT With Contrast The presence of extensive bilateral central pulmonary emboli with filing defects.

On day 3 of hospitalization, an echocardiogram was ordered that revealed right ventricular dysfunction with pulmonary hypertension. Urgent CT with contrast showed extensive bilateral central pulmonary emboli with multiple filing defects distally in the right and left pulmonary arteries extending to the pulmonary artery branches to all lobes of the lung (Figure 3). Interestingly, he maintained adequate arterial oxygen saturation (SaO_2_) on 40% of the fraction of inspired oxygen (FiO_2_) and positive end-expiratory pressure (PEEP) of 5 cm H_2_O. His blood pressure remained to be stable despite multiple vasoconstrictors, and central venous pressure (CVP) was moderately elevated per the pulmonary artery catheter. There was no evidence of cardiogenic shock or lactic acidosis. He had a low clinical indication for urgent tissue plasminogen activator (tPA) after considering the risk and benefits and was instead treated with therapeutic intravenous (IV) heparin. The presenting chief complaint of the near-syncope episode along with the later in-hospital PEA arrest was thought to be related to the hidden extensive bilateral PEs. He remained to be hemodynamically stable overnight.

On day 4-5 of hospitalization, vasoconstrictors were begun to wean off as his blood pressure appeared to be improving. He had maintained stable SaO_2_ as FiO_2_ was further downtitrated to 30%. CVP remained to be in acceptable range per the pulmonary artery catheter. He continued to receive therapeutic heparin treatment. The code status of the patient was changed to do not resuscitate (DNR) by family members after the palliative care team was consulted to discuss goals of care. 

On day 6 of hospitalization, the patient suddenly became hypotensive despite multiple vasoconstrictors, bradycardic, but remained SaO_2_ of 96% on FiO_2_ of 40%. He went into asystole and unfortunately past away as no resuscitative measures were attempted due to DNR status.

## Discussion

Considering PE as a part of the differential diagnosis based on the clinical presentation is extremely important [3]. Over 90% of the patients present with symptoms including dyspnea, chest pain, or tachypnea [4,5]. Other less common symptoms include a clinical impression of deep vein thrombosis (DVT), cough, hemoptysis, fever, tachycardia, or hypoxia [1]. According to data based on a national collaborative study, dyspnea or tachypnea occurred in 92% of the patients found to have PE located in the main or lobar pulmonary arteries [6]. Furthermore, elderly and older patients had similar complaints as age was not found to be a contributing factor.

The importance of being able to recognize venous thromboembolism (VTE)-related risk factors is an essential step in making an accurate diagnosis. Common risk factors include surgery, acute and chronic medical illness, malignancy, known thrombophilia, pregnancy, hormonal replacement therapy, obesity, and immobilization [1]. In addition, certain types of surgical conditions or surgeries, such as hip or leg fracture, hip or knee replacement, major trauma, or spinal cord injury, are the strongest predisposing factors for VTE [7]. The Wells criteria is a frequently used tool both in the outpatient and emergency settings for risk stratification purposes for patients who are suspicious of VTE. It is often used in conjunction with the D-dimer level [3]. A meta-analysis conducted in over 55,000 patients concluded that Wells criteria is more specific and just as sensitive as the clinical impression in diagnosing VTE [8,9]. A score of <2 indicates a low probability, 2-6 shows an intermittent probability, and a score >6 is indicative of a high PE probability. In patients with a low clinical suspicion of PE, the Pulmonary Embolism Rule-out Criteria (PERC) was designed to recognize PE and guide further management [10]. There is a low likelihood of PE if the patient age <50 years, heart rate < 100 beats per minute, oxyhemoglobin saturation ≥ 95%, and no hemoptysis, estrogen use, prior VTE, unilateral leg swelling, and recent hospitalization over the past four weeks. Further imaging is recommended against in patients who fulfill the PERC rule.

Our patient presented to the ED with the initial findings of a near-syncope episode, hypotension, and bradycardia that quickly resolved after fluid resuscitation. PE was not a part of the differential diagnosis until day 3 of hospitalization after his PEA arrest given the overall low clinical suspicion. He was completely asymptomatic with normal hemodynamics while saturation well on room air during the first 2 days of the hospital course. The near-syncope episode was initially thought to be secondary to hypovolemia or potential medication side effects. The Wells criteria was not utilized in the ED due to low PE suspicion despite that he had a likelihood of recent immobilization after his leg amputation. He would have scored a borderline 3 (heart rate of 103 beats per minute and presumptive immobilization within the last 3 days) on the Wells criteria during the initial presentation in hindsight. The score would place him in a moderate risk group with a 16.2% chance of PE in the ED population. A modified Wells criteria would characterize the patient as an unlikely PE candidate. Unfortunately, it was later discovered that the real cause of death was the hidden multiple extensive PE discovered on CT scan after echocardiogram detailed an elevated pulmonary artery pressure with subsequent right heart strain. Interestingly, the PE did not evolve into cardiogenic shock as he maintained adequate oxygenation and hemodynamics on minimal ventilatory settings and pressure support. Thrombolytic and surgical options were never considered given his stable status while receiving therapeutic heparin. He unfortunately past away due to PEA arrest after the family decided to change code status to DNR.

Could the patient’s final destiny be changed if acute PE was high on the differential diagnoses as he set foot into the ED doors? It is a question that is very difficult to answer and is an answer that we will never find out. One would argue that his final destiny could have been the same given the overall clinical impression as the PE was well hidden based on signs and symptoms. Reddy et al. detailed a 27-year-old male who initially presented with back pain later found to have right heart strain on echocardiogram and subsequently diagnosed with PE secondary to hyperhomocysteinemia [11]. Alreshq et al. presented a 92-year-old male with a past medical history of DVT presented with bradycardia later discovered to have PE due to right heart strain on echocardiogram [12]. These examples portray the unpredictability and variation of acute PE presentation that makes timely and proper diagnosis sometimes extremely difficult. 

## Conclusions

Acute PE is a common medical condition that is often associated with high morbidity and mortality. Timely recognition with proper clinical judgment is a gamechanger despite there are situations of atypical presentations. Unfortunately, an accurate clinical decision is extremely difficult, but the consequences of a missed diagnosis could be fatal.
